# Circulating Brain Microvascular Endothelial Cells (cBMECs) as Potential Biomarkers of the Blood–Brain Barrier Disorders Caused by Microbial and Non-Microbial Factors

**DOI:** 10.1371/journal.pone.0062164

**Published:** 2013-04-26

**Authors:** Sheng-He Huang, Lin Wang, Feng Chi, Chun-Hua Wu, Hong Cao, Aimin Zhang, Ambrose Jong

**Affiliations:** 1 Department of Pediatrics, Saban Research Institute of Childrens Hospital Los Angeles, University of Southern California, Los Angeles, California, United States of America; 2 Department of Microbiology, School of Public Health and Tropical Medicine, Southern Medical University, Guangzhou, China; 3 Department of Histology and Embryology, School of Basic Medical Science, Wuhan University, Wuhan, China; 4 Center for Diagnosis and Treatment of Liver Failure, The 302 Hospital, Beijing, China; Washington University, United States of America

## Abstract

Despite aggressive research, central nervous system (CNS) disorders, including blood-brain barrier (BBB) injury caused by microbial infection, stroke, abused drugs [e.g., methamphetamine (METH) and nicotine], and other pathogenic insults, remain the world's leading cause of disabilities. In our previous work, we found that dysfunction of brain microvascular endothelial cells (BMECs), which are a major component of the BBB, could be caused by nicotine, meningitic pathogens and microbial factors, including HIV-1 virulence factors gp41 and gp120. One of the most challenging issues in this area is that there are no available cell-based biomarkers in peripheral blood for BBB disorders caused by microbial and non-microbial insults. To identify such cellular biomarkers for BBB injuries, our studies have shown that mice treated with nicotine, METH and gp120 resulted in increased blood levels of CD146+(endothelial marker)/S100B+ (brain marker) circulating BMECs (cBMECs) and CD133+[progenitor cell (PC) marker]/CD146+ endothelial PCs (EPCs), along with enhanced Evans blue and albumin extravasation into the brain. Nicotine and gp120 were able to significantly increase the serum levels of ubiquitin C-terminal hydrolase 1 (UCHL1) (a new BBB marker) as well as S100B in mice, which are correlated with the changes in cBMECs and EPCs. Nicotine- and meningitic *E. coli* K1-induced enhancement of cBMEC levels, leukocyte migration across the BBB and albumin extravasation into the brain were significantly reduced in alpha7 nAChR knockout mice, suggesting that this inflammatory regulator plays an important role in CNS inflammation and BBB disorders caused by microbial and non-microbial factors. These results demonstrated that cBMECs as well as EPCs may be used as potential cell-based biomarkers for indexing of BBB injury.

## Introduction

Central nervous system (CNS) disorders, including blood-brain barrier (BBB) damage caused by microbial infection (e.g., NeuroAIDS), stroke, drug abuse, brain tumor and neurodegenerative diseases, remain the world's leading causes of disabilities despite aggressive research [1]. Because the brain is the most delicate organ of the body that is protected by the BBB, which constitutes the largest surface area [2], the diseases associated with BBB disorders account for more hospitalizations and prolonged care than almost all other diseases combined. The patients experiencing devastating CNS diseases far outnumber those suffering and dying from all types of systemic cancers or heart diseases [1]. Despite significant advances in highly active antiretroviral therapy (HAART), the prevalence of neuroAIDS has significantly increased [3]. This is mainly due to the inability of antiretroviral drugs to cross the BBB [3] and the role of CNS as the reservoir for HIV-1, which is capable of migrating out of the brain [4]. The incidence of NeuroAIDS is higher or accelerated among the aging populations and drug users [5]–[6]. Over one-third of the entire population will experience a CNS disorder during their lifetime [1]. The incidence of CNS disorders also increases with age. All these factors together, along with the fact that there are no surrogate markers available for the BBB injury, exacerbate the problem of diagnosis/prognosis, prevention and treatment of the CNS disorders.

Quantitative evaluation of the BBB injury has been one of the most challenging issues in the CNS disorders caused by microbial (*e.g*., meningitic pathogens) and non-microbial (*e.g.*, methamphetamine and nicotine) insults [1]–[3], [6]–[18]. Successful isolation and cultivation of BMECs, which are the relevant *in vitro* model of the BBB, has enabled us and others to perform both molecular characterization and genomewide analysis of the pathogenic mechanisms of microbial and non-microbial factor-caused BBB disorders *in vitro* (7–18). However, it is difficult to carry out genomewide, noninvasive evaluation of the *in vivo* BBB injury. A variety of methods have been used to evaluate the function of the BBB *in vivo*. Leakage of peripheral proteins (*e.g*., fibrinogen and albumin) into the CNS has been used to evaluate BBB permeability associated with viral encephalitis and other CNS infection [19]. While these techniques have the advantage of using endogenous proteins, the BBB disruption may not be correlated with the protein levels in CNS due to certain nonspecific effects [19]. Recently, magnetic resonance (MRI)-based molecular imaging technologies have gained increasing attention in neuroscience [20]. Althought an increasing number of synthesized molecular imaging agents have been tested *in vitro*, very few have been validated in the brains of live animals. The major challenges in molecular neuroimaging approaches are the poor ability of delivering agents across the BBB [20]. Additional methods involve the injection of dyes, such as Evans blue and sodium fluorescein (NaFI), into a variety of animal model systems for evaluation of BBB permeability [19]. The major limitation of these techniques is that they cannot be used for humans.

Recently, qualification of circulating endothelial cells (CECs) in peripheral blood has been developed as a novel and reproducible approach for assessing endothelial damage/dysfunction caused by cardiovascular disorders and inflammatory diseases [21]–[24]. The first descriptions of methods used to detect circulating cells in the blood with endothelial characteristics were reported in the mid-1970s [24]. These methods included density centrifugation, vital light microscopy and histologic staining, which did not isolate and identify CECs reliably. It was two more decades before reliable procedures were developed to detect this rare cell population. Currently, the most common CEC qualification procedures include an enrichment step through immunomagnetic separation of cells using magnetic beads coupled to an antibody against an endothelial antigen such as CD146 (endothelial marker) or CD34 (progenitor cell marker) [24]. Among endothelial cells circulating in the blood, some are terminally differentiated mature cells (CECs) while others show progenitor-like phenotype [endothelial progenitor cells (EPCs)], suggesting that EPCs may participate in the generation of new vessels through homing to sites of angiogenesis [24]–[26]. Over the past decade increased CECs have been detected in many pathological conditions, including cancer and heart diseases [21]–[26]. Such cell-based biomarkers, however, are not specifically identified for BBB disorders caused by CNS infection and inflammation. Since the BBB is mainly constituted by the specific endothelial cells, called BMECs, it seems plausible that circulating BMECs (cBMECs) could be biomarkers for BBB dysfunctions. Based on our longstanding interest and studies in the BBB injury and CNS disorders, we have hypothesized that cBMECs, which are endowed with a full-blown BBB phenotype, are dynamically shedding from the brain microvasculature upon pathophysiological changes in the CNS. Circulating BMECs can be monitored by experimental approaches and used as noninvasive blood biomarkers in indexing BBB injury, which is caused by meningitic pathogens and other pathogenic insults. In this report, using animal model systems, we have demonstrated for the first time that BBB injury could be detected by the technologies for characterization and quantification of cBMECs derived from the CNS disorders in mice caused by microbial (gp120 and *E. coli* K1) and non-microbial (methamphetamine and nicotine) insults. Furthermore, we have also demonstrated that alpha7 nAChR, an essential regulator of inflammation [14], plays an important role in cBMEC shedding associated with BBB injury caused by nicotine and meningitic *E. coli* K1.

## Results

### Whole Blood Magnetic Affinity Isolation and Immune-identification of cBMECs as Well as Related ECs

**Figure 1:**
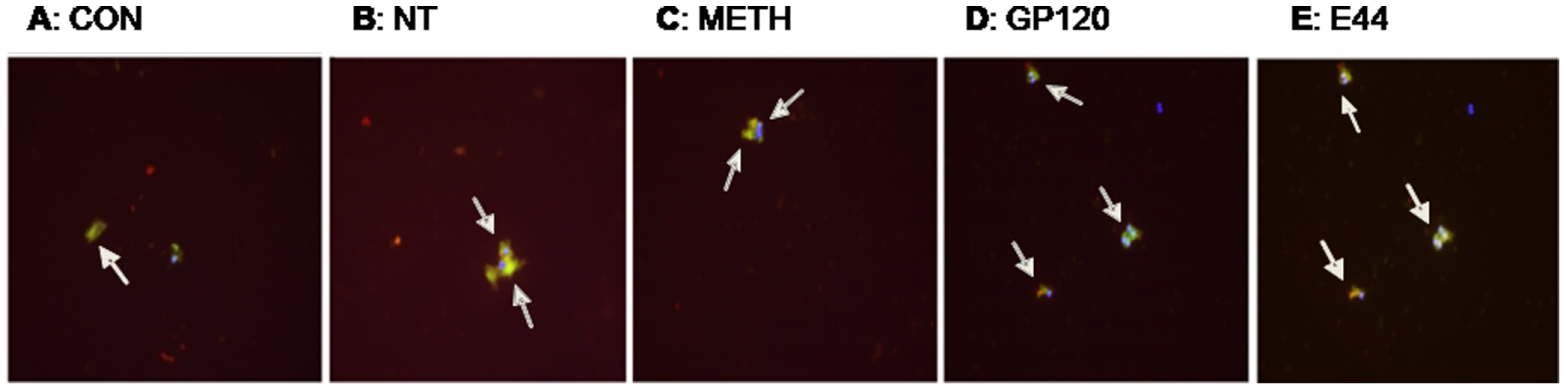
Triple staining (TS) of murine cBMECs (A–E) isolated by the use of UEA magnetic beads. TS was done by DAPI (blue)/antibodies against CD146 (FITC/green) (for EC) and S100B (for brain) (rhodamine/red) (**A–D**: **cBMECs**, CD146+/S100B+/DAPI+). Cells indicated with arrows are cBMECs (**A–E**) from mice treated with PBS (**A**: Control), NT (**B**), METH (**C**), gp120 (**D**), and meningitic *E. coli* K1 E44 (**E**).

**Figure 2:**
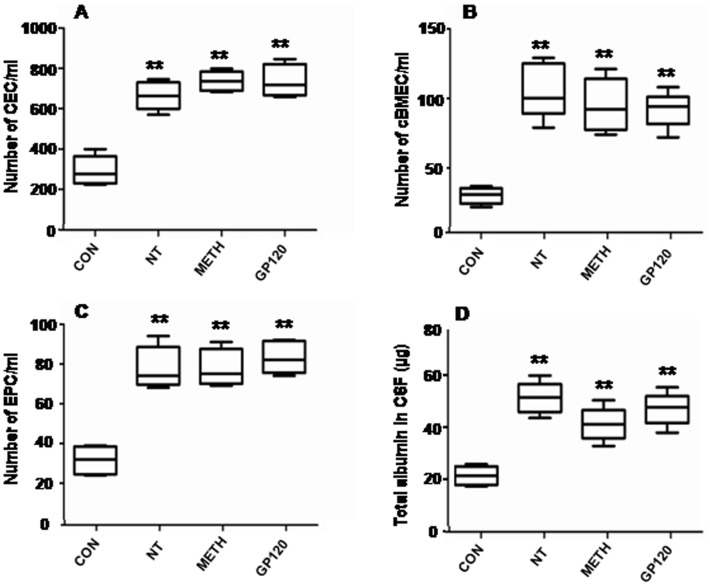
BBB disorders induced by NT, METH, and gp120. Triple staining (**TS**) of murine cBMECs and EPCs was done by DAPI (blue)/antibodies against CD146 (FITC/green) (for EC) and S100B (for brain) (rhodamine/red) (**cBMECs**, CD146+/S100B+/DAPI+) or CD133 (for PC/rhodamine/red) (**EPCs**, CD146+/CD133+/DAPI+). cBMECs and EPCs were counted with six random fields. Number of total ECs (CEC) (**A**), cBMECs (**B**) and EPCs (**C**) in peripheral blood (ml). Quantification of albumin in CSF (**D**). (**P<0.001).

In order to determine whether cBMECs as well as related ECs are present in peripheral blood and can be used as noninvasive blood biomarkers in cellular indexing of BBB injury caused by microbial and non-microbial pathogenic insults, magnetic bead extraction (MBE) was used to quantitatively evaluate BBB injury in mice caused by NT, METH, gp120 and E44through measuring of cBMECs derived from the brain. Using UEA-I coated beads, ECs in peripheral mouse blood were evaluated as described previously [27]. Total ECs or CECs (CD146+/DAPI+), cBMECs (CD146+/S100B+/DAPI+) and EPCs (CD146+/CD133+/DAPI+) were identified based on their S100B [28] (brain marker)^+^/CD146 [21]–[22] (EC marker)^+^/CD133+ (PC marker)[29]–[30]/DAPI (nuclei)^+^phenotype (Figure 1A–E). Flow cytometry [9] was also used for detection of cBMECs in peripheral mouse blood by using directly conjugated antibodies against CD45-Cy5 (a marker for haematopoietic cells), CD31-APC (a marker for ECs), CD34-FITC (a marker for Hematopoietic stem cell), and GGT-FITC (gamma-glutamyltranspeptidase) (brain capillaries). There was a good agreement between the two methods for cBMEC quantification (data not shown). Our studies concurred with the literature that there were low blood levels of CECs (<400/ml) (Figure 2A) and EPCs (<40/ml) (Figure 2C) in the control group of animals. We have demonstrated for the first time that very small numbers of cBMECs (<35/ml) could be isolated from the total population of CECs and that significant changes in the levels of cBMECs and EPCs in the peripheral bloodstreams were induced in the animals treated with microbial and non-microbial factors (Figure 2).

### Levels of cBMECs and EPCs are Significantly Increased in Mice Treated with Nicotine, METH and HIV-1 gp120

**Figure 3:**
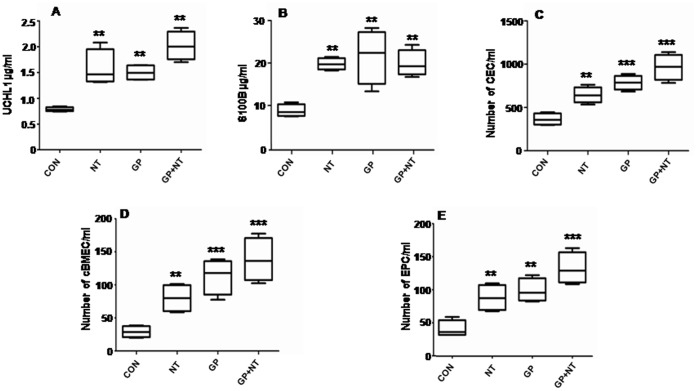
Blood levels of UCHL1 (A), S100B (B), CECs (C), cBMECs (D) and EPCs (E) in mice treated with PBS (CON), nicotine (NT), gp120 (GP) and nicotine+gp120 (NT+GP). Bars denote mean values, and error bars describe SEM. **P<0.01 & *** P<0.001 compared with the control (PBS).

The levels of CECs, cBMECs and EPCs are significantly higher for the mice treated with drugs (METH or nicotine) and gp120 when compared to the control (Figure 2A–C), suggesting the involvement of systemic inflammatory response (CEC), the BBB injury (cBMECs) and mobilization of EPCs. Nicotine was able to enhance HIV-1 gp120-induced cBMEC shedding (Figure 3D). Interestingly, increased numbers of EPCs are correlated with EPC cluster formation induced by nicotine, METH and gp120 (Figure S1). In order to confirm the reliability of the MBE-based method, quantitative evaluation of METH-, NT-, and gp120- caused BBB injury was carried out by quantification of albumin in CSF, which have been extensively used for assessing BBB disruption [31]–[33]. As shown in Figure 2D, METH, nicotine, and gp120 were able to significantly increase the BBB permeability to albumin. The similar results were obtained with the Evans blue assays (data not shown), suggesting that changes in the BBB permeability were correlated with cBMEC shedding. These findings demonstrate that quantification of cBMECs and EPCs by MBE is feasible for evaluating the BBB disruption caused by pathogenic insults.

### Changes in cBMEC Levels are Correlated with Alterations of Molecular Markers S100B and UCHL1

To further validate the biological relevance of the cell-based biomarkers, the correlation of cBMECs and molecular biomarkers (S100B and UCHL1) was tested in the mouse model as described in Methods and Materials. UCHL1, which has a more specific tissue distribution than S100B, is found more exclusively in neurons and is associated with traumatic brain injury [34], but it was unclear whether it could play a role in BBB disorders caused by microbial factors (e.g., gp120) and drugs of abuse. To determine if there was a correlation between cBMECs and S100B/UCHL1, and if UCHL1 could be used as a novel molecular marker for BBB disorders caused by drugs of abuse (*e.g*., nicotine) and the HIV-1 proteins such as gp120, the animal treatment and cBMEC/EPC quantification were performed as described in the first experiment. Serum levels of molecular markers were determined by ELISA using antibodies and antigens from Creative Biomart (New York, NY) (S100B) and ProteinTech (Chicago, IL) (UCHL1). Our results showed that nicotine and gp120 were able to increase blood levels of both molecular (UCHL1 and S100B) and cellular (cBMECs and EPCs) markers (Fig. 3A–E), suggesting that UCHL1 is a potential new biomarker for BBB disorders caused by drugs of abuse and microbial factors. The combination of treatment with nicotine and gp120 significantly enhanced the levels of biomarkers when compared to the groups treated with nicotine and gp120 alone, indicating that nicotine was potentiating the toxic effects of gp120.

### In vivo-in vitro Correlation of cBMEC Shedding

**Figure 4:**
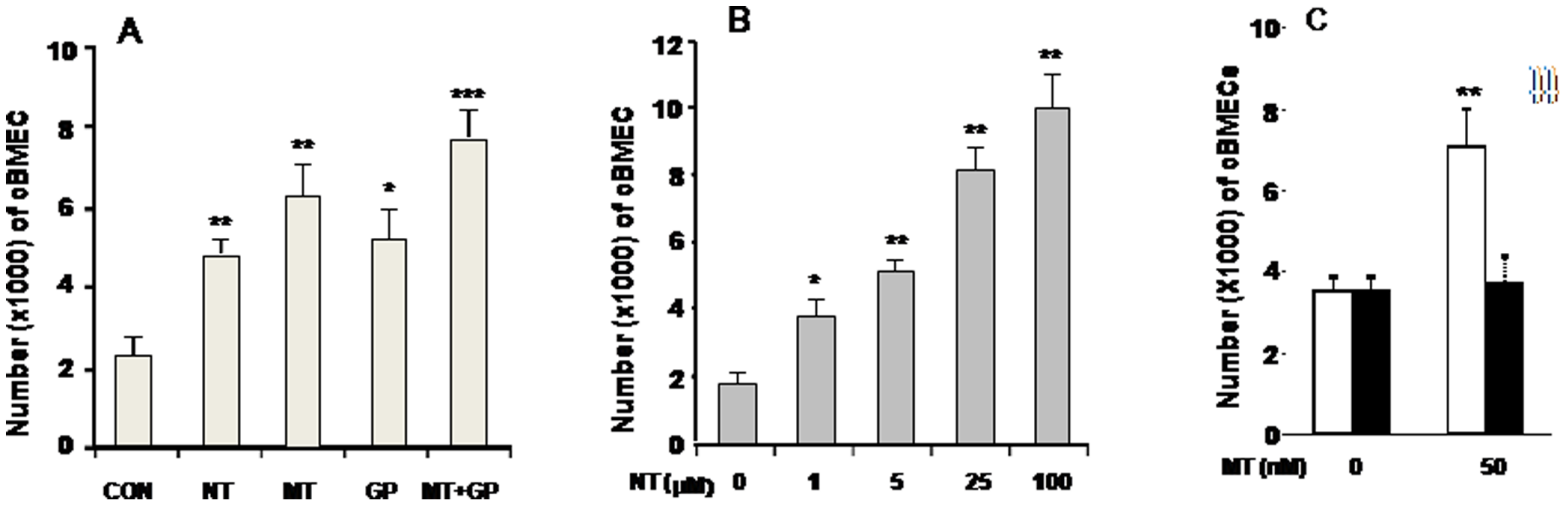
Enhancement of cBMEC shedding *in vitro* by nicotine (NT), METH (MT) and gp120. ***A***. Shedding of cBMECs from BMEC monolayers in the upper chambers of Transwells after exposure to nicotine (10 µM), METH (10 nM) and gp120 (50 ng/ml) for 36 h. ***B***. cBMEC shedding from the WT BMEC monolayers after exposure to different doses of nicotine. ***C***. cBMEC shedding from WT (white column) and KO (black column) BMEC monolayers treated with METH. Bars denote mean values, and error bars describe SEM. ^*^ *p*<0.0 5; **P<0.01.

The next set of experiments were designed to address the in vivo-in vitro correlation of cBMEC shedding. The approach exploited an in vitro model in which BMECs were treated with microbial (gp120) and non-microbial (NT and METH) factors likely to be important in the pathogenesis of BBB disorders. Specifically, BMECs were cultured in the presence or absence of gp120, NT, METH and gp120 plus NT or METH (MT). Treatment was followed by counting of cells in the lower chambers for assessment of cBMEC shedding. As seen in Figure 4A, NT, METH and gp120 could significantly induce cBMEC shedding than the treatment with medium alone (CON). Moreover, significantly higher levels of cBMECs were detected in the combination of gp120 with NT or METH. NT could significantly increased cBMEC shedding in a dose-dependent manner (Figure 4B). In order to establish *in vitro* models for examining the role of α7 nAChR in cBMEC shedding, wildtype (WT) and α7 nAChR knockout (KO) BMECs were isolated and purified from the brains of 10-day-old wildtype (α7^+/+^) and α7-deficient mice (α7^−/−^) using UEA I lectin-coated beads as described previously [14], [35]. Most interestingly, METH-induced cBMEC shedding was abolished in α7 nAChR-deficient BMECs when compared to the wildtype cells (Figure 4C).

### α7 Deficient Animals are Defective in BBB Disorders Caused by Microbial (Meningitic *E. coli* K1) and Non-microbial (NT) Factors (Fig. 5A–D)

**Figure 5:**
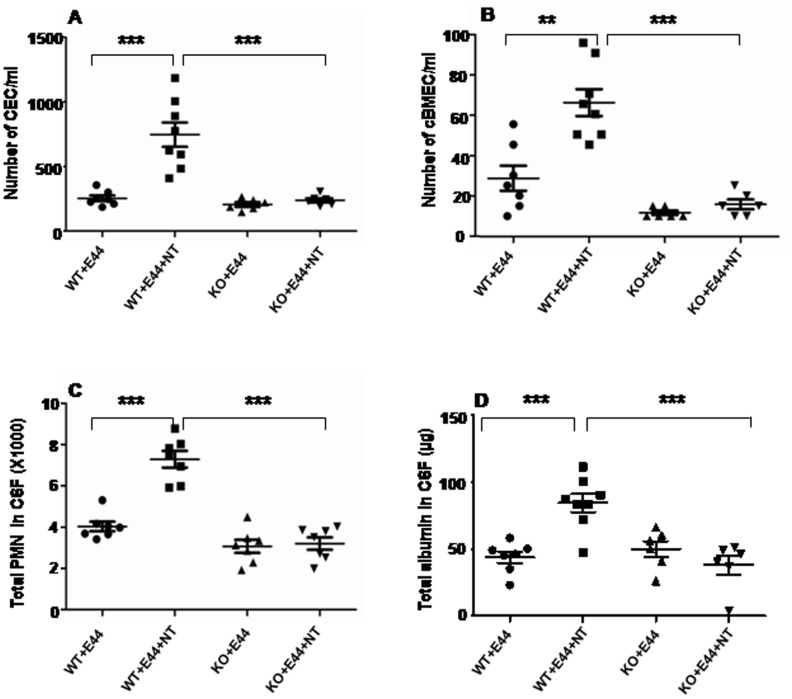
Effects of genetic blockage of α7 nAChR on nicotine-increased BBB permeability and E44 transcytosis. Triple staining (TS) of murine cBMECs isolated by the use of magnetic beads coupled with UEA-I, which specifically binds to EC [35]. CEC and cBMECs were isolated from wildtype (WT) and α7 deficient (KO) murine pups treated with nicotine (NT), E44 or NT plus E44. Cells without treatment were used as a control. TS was done by DAPI (blue)/antibodies against CD146 (FITC/green) (A: CEC) and S100B (for brain) (rhodamine/red) (B: cBMECs, CD146+/S100B+/DAPI+) or CD133 [for Progenitor ECs(PEC)/rhodamine/red] (PEC: CD146+/CD133+/DAPI+)(Figure S1). CECs and cBMECs were counted with six random fields. (*P<.05; **P<0.01; ***P<0.001). CNS inflammation and BBB injury were further confirmed by quantification of PMN (C) and albumin (D) in CSF, which have been extensively used for assessing BBB disruption [31].

As α7 nAChR plays an important role in CNS inflammation induced by microbial and non-microbial factors [10], [14]–[15], we have proposed that turnover and shedding of BMECs could be regulated by α7 nAChR. To test this hypothesis, the correlation of α7 nAChR with cBMEC shedding and CNS inflammation induced by meningitic *E. coli* K1 (E44) and nicotine was examined in the gene knockout mouse model of α7 nAChR as described in Methods and Materials. In this study, wildtype (α7^+/+^) and KO (α7^−/−^) neonatal (10 day-old) mice were intraperitoneally injected with E44 after treatment with nicotine for 3 days. As shown in Figure 5, nicotine could significantly increase shedding of both CEC (5A) and cBMECs (5B) but could not induce any changes in KO mice treated with E44 alone or combined with nicotine (P<0.01). The levels of cBMECs were moderately increased in the WT mice treated with E44 alone, while the combination of E44 with nicotine greatly enhanced cBMEC shedding, suggesting that α7 nAChR plays an essential role in the synergistic effects of nicotine on BBB disorders caused by E44. Similarly, the PMN counts (Figure 5C) and albumin extravasation (Figure 5D) in CSF were significantly reduced in KO mice as compared to wildtype animals (P<0.001). Nicotine was only able to enhance PMN transmigration and albumin extravasation in wildtype mice as compared to corresponding controls (P<0.001), suggesting that α7 nAChR also contributes to the correlation of CNS inflammation with cBMEC shedding. Taken together, these data suggested that α7 nAChR could play an essential role in regulation of CNS inflammation and cBMEC shedding induced by microbial and non-microbial factors.

## Discussion

**Figure 6:**
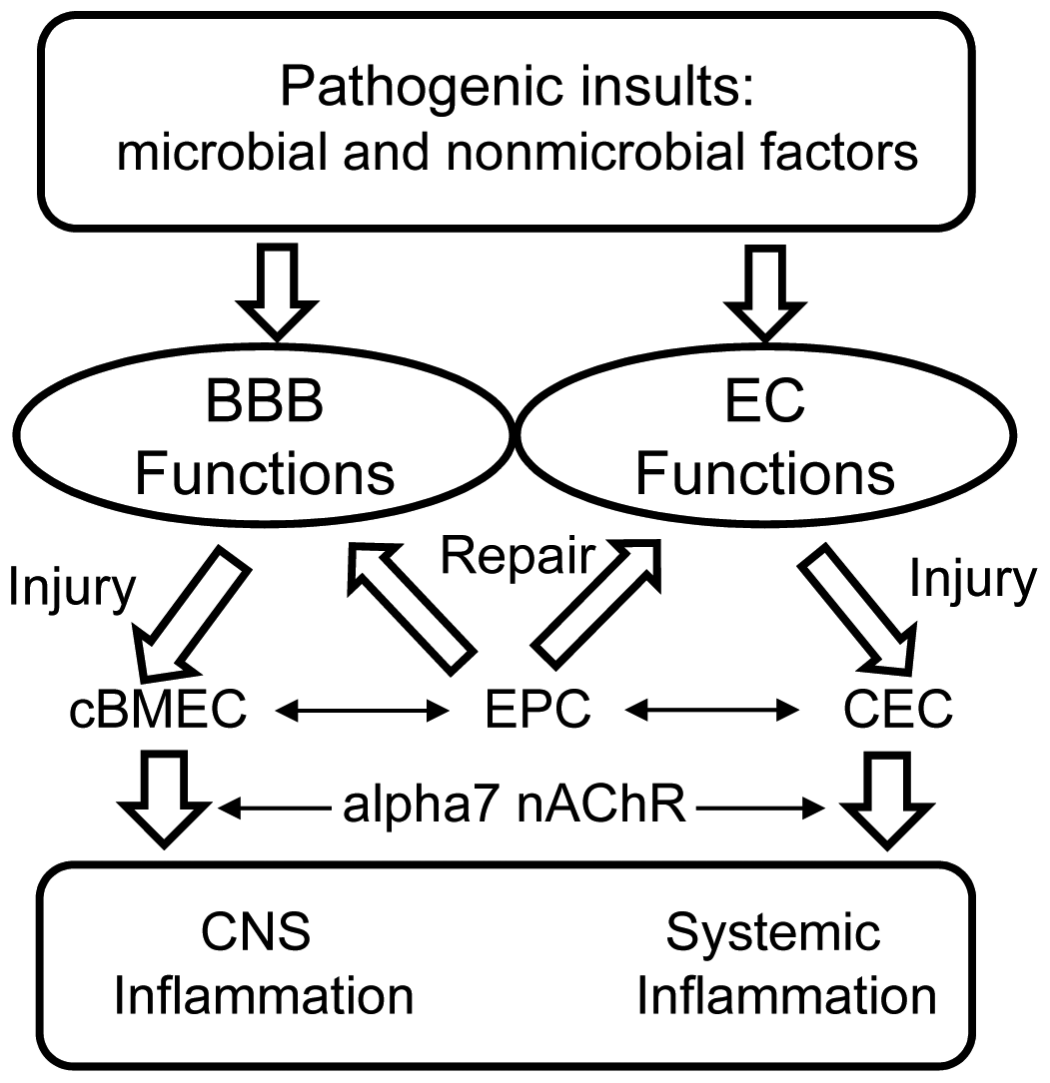
Role of cBMECs and EPCs in physiology and pathology of the BBB. During BBB vascular turnover BMECs might be replaced by proliferation of adjacent cells or by maturation of circulating endothelial progenitors (EPCs) generated in the bone marrow. Circulating endothelial cells (CECs) and BMECs (cBMECs) with a mature phenotype, derived from systemic and BBB vessel turnover, respectively, are increased in patients with systemic inflammation and BBB disorders. The role and the frequency of marrow-derived circulating EPCs may vary in different types of CNS inflammation and in different phases of BBB disorders. In addition to cBMECs and EPCs, cerebral angiogenesis might be modulated by some other specialized cells such as astrocytes and pericytes.

We have developed a new model for the discovery of cell-based BBB biomarkers (Figure 6). Our studies have suggested for the first time that cBMECs can be detected and be used as a cellular index of the BBB damage caused by microbial (*E. coli* K1 and gp120) and non-microbial (nicotine and METH) pathogenic insults. The inflammatory response may be regulated by α7 nAChR. It has been a very challenging issue to directly make the quantification of the BBB injury caused by various pathogenic insults [8]. Most biomarker research into CNS disorders has focused on neuronal damage not on BBB injury, because neuronal sensitivity to certain pathogenic insults is region- and disease-specific [36]. Therefore, much previous research on brain injury has focused on biomarkers that measure neuronal damage. There are a number of clinical advantages for shifting the focus on BBB injury. Firstly most CNS diseases are accompanied by increased BBB dysfunction. Secondly BBB injury does occur concomitantly with the pathogenic insult, but neuronal damage may develop slowly or after a delay. The early detection of BBB disorders offers a window opportunity for neuroprotective intervention. Another advantage is that the cell-based biomarkers cBMECs along with the single cell profiling (SCP) approaches will make the diagnosis of CNS disorders much easier and acceptable. Recent advances in biotechnologies for proteomic and genomic analysis at single-cell resolution enable a global novel understanding of complex biological processes [37]–[41]. The SCP approaches will allow the study of multiple genes/proteins or entire genomes/proteomes of cBMECs. Considering these advantages, the current studies are primarily designed to lay foundations for the use of cBMECs as cellular biomarkers of the BBB injury, which contributes to various CNS disorders. In the current report, we demonstrated that cBMECs could be used as cell-based biomarkers for BBB disorders caused by microbial (e.g., gp120 and meningitic *E. coli* K1) and non-microbial (e.g., nicotine and METH) factors.

Interestingly, increased numbers of EPCs are correlated with EPC cluster formation induced by nicotine, METH and gp120. EPCs, derived from bone marraw, are capable of homing to damaged endothelium and furthermore contribute to re-endothelialization and neovascularization [42]–[43]. A correlation of increased EPC number and cluster formation in peripheral blood is observed in BBB injury caused by pathogenic insults on the basis of our study, consistent with the published reports on EPCs mobilized by myocardial ischemia and EPC cluster formation in cerebral small vessel disease [44]–[45]. There are two mechanisms by which endothelial repair occurs have been recently identified [46]. The lost and damaged cells can be replaced by local replication of adjacent mature endothelial cells. However, if local replication were the dominant mechanism of endothelial repair, it would rapidly lead to loss of endothelial integrity. More recently, it has become clear that maintenance and repair of the endothelium by circulating EPCs is an alternative mechanism. These circulating cells in the peripheral blood are derived from the bone marrow, and can differentiate into mature cells with endothelial characteristics. Our findings suggest that EPCs may actively participate in the repair of BBB.

BMECs may have antigenic overlap with non-BMECs. To determine the total cBMEC number but not a subpopulation, it is essential to select markers that are specifically and constantly expressed by all cBMECs. So far, there are no such markers that meet these criteria. Therefore, the assays depend on multiple characteristics to detect cBMECs as well as EPCs. UEA-I (for EC), CD146 (for EC), CD133 (for PC), GGT (for brain) and S-100B (for brain) are used as detection markers. UCHL1 (also known as PGP9.5), which is a component of the ubiquitin proteosome system [47]–[48], was first detected as “brain-specific protein” in 1981 [48]. This protein has a more specific tissue distribution than S100B and is found more exclusively in neurons [34]. Increased expression of UCHL1 is associated with mechanical stress-caused vascular damage [49], oncogenesis [50] and traumatic brain injury [34]. UCHL1 acts as an oncogene and is found to be related to lymph node metastasis in colorectal cancer [50]. It belongs to the family of deubiquitinating enzymes (DUBs), which constitute the ubiquitin-dependent proteolytic system (UPS). DUBs are emerging as important regulators of many pathways contributing to regulation of both oncogenes and tumor suppressors [51]. Cancer can be promoted by both overexpression and loss of function of DUBs. The metabolic dysregulation of DUBs may contribute to oncogenesis and inflammatory diseases. However, it was unclear whether UCHL1 was associated with BBB injury caused by microbial factors and drugs of abuse. Our data showed that elevated serum levels of UCHL1 were significantly correlated with increased cBMECs in the animals treated with gp120 and nicotine, suggesting that this protein could be used as a new molecular biomarker for BBB injury caused by microbial and non-microbial factors. Recent studies suggest that UCHL1 acts a component of the UPS and contributes to injury-caused vascular remodeling through modulation of NF-κB activity and related signaling pathways [49], [52]. UCHL1 may also contribute to regulation of BBB integrity. While UCHL1 is more specific than S100B, it is also present in non-BMEC vascular ECs [49], [52]. SCP may offer new approaches for identification of specific BBB biomarkers through genomewide analysis of cBMECs.

Our previous studies demonstrated that both microbial (e.g., *E. coli* K1) and non-microbial (e.g., nicotine) factors could up-regulate α7 nAChR and that CNS inflammation induced by these pathogenic insults could be blocked by α7 antagonist-mediated inhibition and genetic knockout of the α7 gene [10], [14]–[15]. In concurrence with these findings, α7 nAChR could be up-regulated by METH and gp120, which are involved in the pathogenesis of HIV-associated neurocognitive disorder (HAND) [53]–[56]. In this report, we have established that α7 nAChR plays an important role in regulation of BBB integrity in the mouse model. The pathogenic insult-induced cBMEC shedding, which is correlated with increased BBB permeability, is significantly reduced in the α7-deficient mice. These data suggest that up-regulation of α7 nAChR is detrimental to the BBB integrity and function.

The precise mechanism responsible for the pathogenic insult-mediated increase in BBB permeability and cBMEC shedding during CNS inflammation is unknown. Although it is well-known that proinflammatory factors promote increased BBB permeability, it is unclear how the production of these factors is regulated during CNS disorders. Our previous studies showed that α7 nAChR could directly or indirectly upregulate proinflammatory factors (IL-1β, IL-6, TNFα, MCP-1, MIP-1α, RANTES, CD44 and ICAM-1), significantly enhance PMN transmigration into CSF and has a detrimental effect on the permeability of the BBB in the early stages of meningitic infection [14]. Calcium signaling mediated by α7 nAChR is the major regulatory pathway for the CNS inflammatory response to meningitic *E. coli* infection and nicotine exposure. Using the α7 KO mouse model, we demonstrated that decreased cBMEC shedding was correlated with CNS inflammatory response (*e.g*., decreased PMN recruitment and albumin leakage into CSF) when compared to that in the wildtype animals. These findings provide insight into an element of host defense previously unknown to contribute to the BBB integrity and cBMEC shedding, but the implications of the cholinergic α7 nAChR pathway for the pathogenesis and therapeutics of BBB disorders and CNS inflammation remain to be explored. Alpha7 nAChR has been found to be able to mediate SLURP (secreted mammalian Ly-6/urokinase plasminogen activator receptor-related protein)-1-upregulated NF-κB through both ionic events (calcium signaling) and activation of protein kinases [57]. Both UCHL1 and S100B are shown to be involved in regulation of NF-κB [49], [52], [58]. It is likely that α7 nAChR-mediated NF-κB signaling may be involved in regulation of both the molecular (UCHL1 and S100B) and cellular (cBMEC shedding) biomarkers during various CNS disorders.

In conclusion, the blood levels of cBMECs as well as EPCs positively correlate with BBB injury and host inflammatory response during CNS inflammation induced by microbial and non-microbial factors. These results enlighten the potential of these noninvasive cell-based biomarkers in indexing BBB injury and optimize therapeutic options.

## Materials and Methods

### Ethics Statement

This study was performed in strict accordance with the recommendations in the Guide for the Care and Use of Laboratory Animals of the National Institutes of Health. Our protocols were approved by the Institutional Animal Care and Use Committee (IACUC) of The Saban Research Institute of Children’s Hospital Los Angeles (Permit number: A3276-01). All surgery was performed under anesthesia with ketamine and lidocaine, and all efforts were made to minimize suffering.

### Chemicals and Reagent

Nicotine tartrate (NT) and methamphetamine (METH) were purchased from Sigma-Aldrich (St. Louis, MO). Dynabeads M-450 Tosylactivated was obtained from Invitrogen (Carlsbad, CA). Ulex europaeus I (UEA I) lectin and mounting medium with 4',6-diamidino-2-phenylindole (DAPI) were purchased from Vector (Buringame, CA). Gp120 was purchased from Immunodiagnostics (Bedford, MA). Serum levels of molecular markers were determined by ELISA using antibodies and antigens from Creative Biomart (New York, NY) (S100B) and ProteinTech (Chicago, IL) (ubiquitin C-terminal hydrolase 1, UCHL1). All primary antibodies (Ab) were purchased from the commercial sources: a rabbit anti-α7 nAChR Ab from Genescript (Piscataway, NJ); a rat anti-mouse Ly-6G (Gr-1) Ab; a mouse anti-CD44 Ab (sc-7297), a rabbit anti-β-actin (sc-7210), and a rabbit anti-GGT Ab (sc-20638) from Santa Cruz Biotechnology (Santa Cruz, CA); a rat anti-mouse Ly-6G (Gr-1) Ab FITC-conjugated and an anti-mouse CD146 Ab FITC-conjugated from eBiosciences (San Diego, CA), a rabbit anti-S100B Ab rhodamine-conjugated from BD Biosciences, and a rabbit anti-CD133 Ab rhodamine-conjugated from Abbiotec (San Diego, CA). Transwell filters (3 µm pore size, 6.5 mm diameter), blood plates and CBA assay kit were purchased from BD Biosciences (San Jose, CA).

### Animal Model and Treatment Protocol

All animal experiments were performed using C57BL/6J mice after approval from the IACUC of The Saban Research Institute of Children’s Hospital Los Angeles. Heterozygous (+/−) α7-deficient mice with the C57BL/6J background (B6.129S7-Chrna7^tm1Bay^/J) were purchased from Jackson Laboratory (Bar Harbor, ME). Genotypes of α7^+/+^ mice (WT mice), α7^−/−^ mice (KO mice) and heterozygous α7^+/−^ mice were determined according to the PCR protocol provided by the vendor. The animals were used in transgenic breeding at 8 weeks of age for optimum reproductive performance. Male heterozygous (+/−) and female homozygous (−/−) were used in breeding. The average litter size for neonatal mice was 6–8. Age- and sex-matched mice were used in all experiments. Three experiments were carried out. In Experiment 1, WT mice (4 week-old) were divided into 4 groups (I: Control treated with PBS; II: NT; III: METH; and IV: gp120) (n = 5). Two groups (II and III) of animals were exposed to low dose (1.5 mg/kg/day) of NT (oral delivery) for 3 days (twice per day) or gradually increased doses (2, 4, 6, 8, 10, 10, 10,10, 10, 10 mg/kg from day1 to day10) of METH [intraperitoneal (i.p.) injection] for 10 days as described previously [59]–[60]. The animals in Group IV received daily injections from tail veins (50 ng/mouse) of endotoxin-free recombinant HIV-1 gp120 for 2 days as described previously [61]–[62]. The doses of drugs are relevant to the clinical settings of smokers [59], METH abuse [60] and HIV/AIDS (gp120 in serum 12–92 ng/ml) [63]. To determine if UCHL1 could be used as a novel molecular marker for BBB disorders caused by NT and HIV-1 proteins, Experiment 2 was carried out. Mice (WT) were divided into 4 groups **(I**: Control treated with PBS; **II**: NT; **III**: gp120; **IV**: NT+gp120; n = 4). The animal treatment was performed as described in the first experiment. Serum levels of molecular markers were determined by ELISA using antibodies and antigens from Creative Biomart (New York, NY) (S100B) and ProteinTech (Chicago, IL) (UCHL1). In Experiment 3, the role of α7 nAChR in cBMEC shedding was tested in the neonatal mouse model of *E. coli* K1 (E44) meningitis using WT (α7^+/+^) and KO (α7^−/−^) mice. Animals (15 to 20-days old were divided into four groups (I: WT infected with E44; II: WT exposed to NT and infected with E44; III: KO infected with E44; and IV: KO exposed to NT and infected with E44) (6–8 mice/per group). The animals (II & IV) were exposed to NT as described in Experiment 1. After NT exposure, all mice received *E. coli* K1 strain E44 (2×10^5^ CFU) by intraperitoneal injection. Eighteen hours after *E. coli* inoculation, the animals were anaesthetized with ketamine and lidocaine, and blood samples were collected from heart puncture for bacterial culture using sheep blood plates. After perfusion from heart puncture with 20 ml PBS [64], the skull was opened. CSF samples were collected as described previously [32], [65]. For bacteria counting in CSF, 20 µl CSF samples were taken and diluted for bacterial culture with blood plates. For PMN counting in CSF, 50 µl CSF samples were stained with a FITC-conjugated rat anti-mouse Ly-6G (Gr-1) antibody and counted under fluorescence microscopy. Albumin concentrations in CSF samples were determined using a mouse Albumin ELISA kit from Bethyl laboratories (Montgomery, TX) according to the manufacturer.

### Isolation and Counting of Mouse cBMECs

Mouse cBMECs and BMECs were isolated with Ulex europaeus I (UEA I) lectin-coated Dynabeads as described previously [35]. The beads were prepared according to the manufacturer’s instructions (Invitrogen) and resuspended in Hanks' balanced salt solution (HBSS, Invitrogen Corp., Carlsbad, CA, USA) plus 5% fetal calf serum (HBSS+5%FCS) to a final concentration of 4×l0^8^ beads/ml. Mouse CECs, cBMECs and EPCs in whole blood were affinity captured at 4°C with UEA-I-coated Dynabeads. To eliminate non-specific cell binding to the beads, the cell suspensions were flushed through the pipette tip during the washing steps and then suspended in PBS. The cells were transferred to glass splices to by cytospin for staining and counting under a fluorescence microscope. Total ECs or CECs (CD146+/DAPI+), cBMECs (CD146+/S100B+/DAPI+) and EPCs (CD146+/CD133+/DAPI+) were identified based on their S100B [28] (brain marker)^+^/CD146 [21]–[22] (EC marker)^+^/CD133+ (PC marker)(29–30)/DAPI (nuclei)^+^phenotypes.` Alternatively, flow cytometry was used for detection of cBMECs in peripheral mouse blood using the following anti-mouse antibodies: CD45-Cy5 (a marker for haematopoietic cells), CD31-APC (a marker for endothelial cells) and CD34-FITC (a marker for Hematopoietic stem cell). A rabbit anti-GGT (gamma-glutamyltranspeptidase) antibody and FITC-conjugated anti-rabbit IgG antibody were used to stain GGT, a marker for brain capillaries. Flow cytometry was carried out as described previously [9] using a FACSCalibur flow cytometer (BD Biosciences) and acquired data analyzed with CellQuest flow cytometry analysis software, with analysis gates designed to remove residual platelets and cellular debris. cBMECs derived from the BBB were identified based on their GGT^+^CD31^+^CD45^–^ phenotype. BMECs were prepared from mouse brain tissues as described previously [14], [35]. Briefly, the mouse (10-day-old) cerebral cortex specimens devoid of large blood vessels were used for isolation of crude microvessels, which were further digested with collagenase (0.1 U/ml), dispase (0.8 U/ml) and DNase I (10 U/ml). Microvascular capillaries were isolated by absorption to Ulex-coated beads. The confluent BMEC monolayer displays a cobblestone appearance when grown on collagen-coated surfaces. The cells were positive for CD146 [22], demonstrating their endothelial origin, and also expressed S100B [28] and GGT [66], indicating their brain origin. The cells also exhibited the typical characteristics for brain endothelial cells expressing tight junctions and a polarized transport of rhodamine 123, a ligand for P-glycoprotein [67].

### Transwell Assays of cBMEC Shedding

To further investigate cBMEC shedding, the double-chamber Transwell-based *in vitro* BBB model has been used. BMECs were isolated from WT and α7 nAChR KO mice as described in our recent publication [14]. BMECs were cultured on collagen-coated Transwell polycarbonate tissue-culture inserts with a pore diameter of 12 µm (Corning Costar) for 5 days [68]. BMECs were polarized and exhibited a trans-endothelial electrical resistance (TEER) of 200–250 Ω cm^−2^, as measured with an Endohm volt/ohm meter in conjunction with an Endohm chamber (World Precision Instruments) as described previously [68]. After exposure of the BMEC monolayer in the upper chamber to low doses of METH (10 nM) [69], NT (10 µM) [10], gp120 (50 ng/ml) [69] and METH (10 nM)+gp120 (50 ng/ml) for 36 h, the shed cBMECs in the lower chambers were counted under the microscope. Simultaneously, the integrity of the BMEC monolayer was assessed by measurement of the TEER. Three measurements were made at each time-point for each sample.

### Statistical Analysis

For the analysis of the *in vitro* data, ANOVA and covariates followed by a multiple comparison test such as the Newmann-Keuls test were used to determine the statistical significance between the control and treatment groups. Software GraphPad Prsim 5.0 was used for analysis of data from animal experiments. P<0.05 was considered to be significant.

### Database

The protein access codes in Swissprot database are listed as follows: α7 nAChR, *Mus muscularus*, Q9JHD6; CD31, *Mus muscularus*, Q08481; CD34, *Mus muscularus*, Q64314; CD45, *Mus muscularus*, P06800; CD146, *Mus muscularus*, Q8R2Y2; S100B, *Mus muscularus*, P50114; GGT, *Mus muscularus*, Q60928; UCHL1, *Mus muscularus*, P09936.

## Supporting Information

Figure S1 **Triple staining (TS) of murine EPCs (A–E) isolated by the use of UEA magnetic beads.** TS was done by DAPI (blue)/antibodies against CD146 (FITC/green) (for EC) and CD133 (for PC/rhodamine/red) (**EPC**, CD146+/CD133+/DAPI+). Cells indicated with arrows are EPCs (**A–D**) from mice treated with PBS (**A**: Control), NT (**B**), METH (**C**), and gp120 (**D**).
(TIF)
