# Supplementary figures and images for: Circulating Brain Microvascular Endothelial Cells (cBMECs) as Potential Biomarkers of the Blood–Brain Barrier Disorders Caused by Microbial and Non-Microbial Factors
Source: PLoS One. 2013 Apr 26;8(4):e62164. doi: 10.1371/journal.pone.0062164 (PMC3637435; doi:10.1371/journal.pone.0062164)

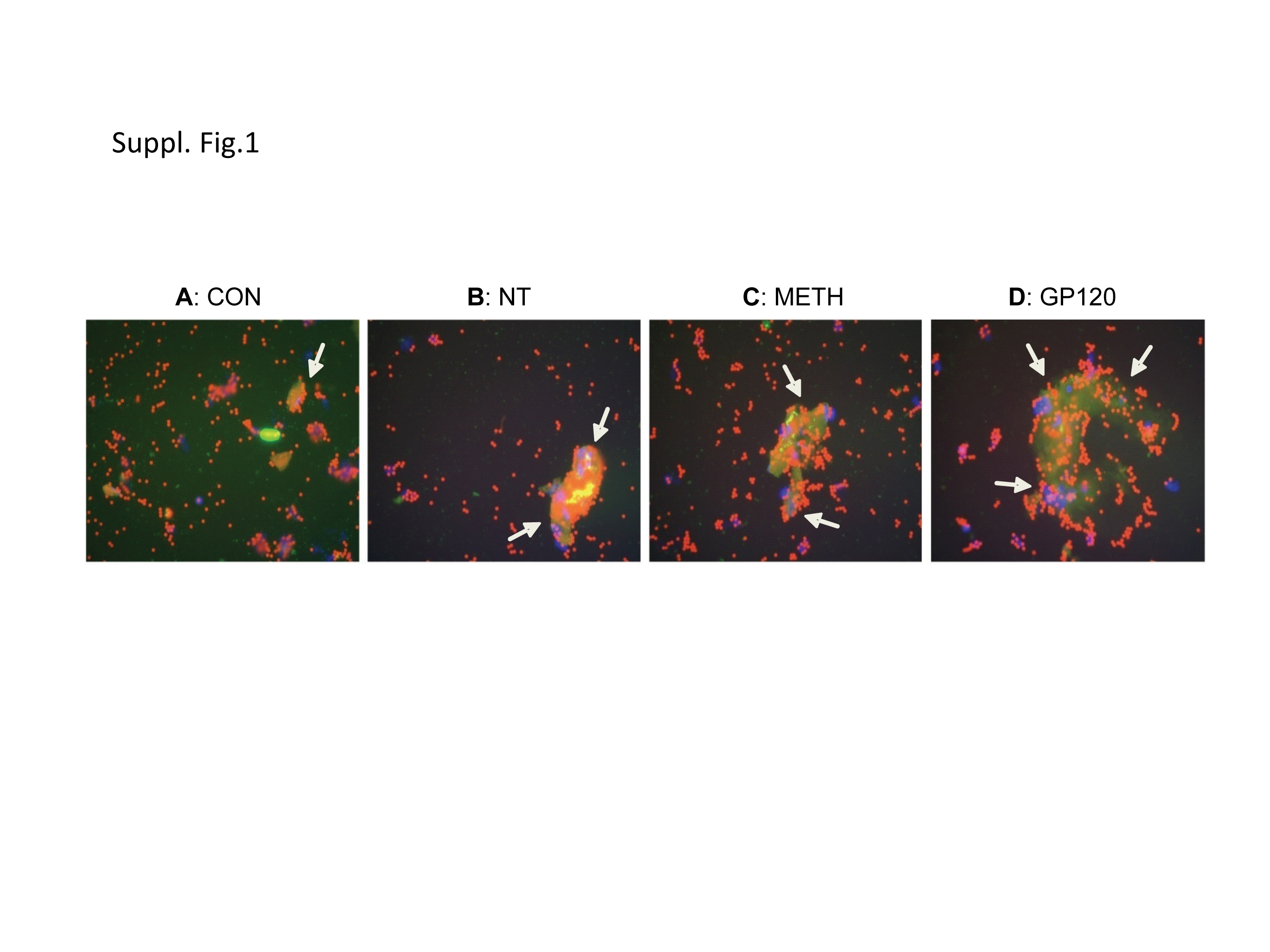

Supplement: Figure S1 — Triple staining (TS) of murine EPCs (A–E) isolated by the use of UEA magnetic beads. TS was done by DAPI (blue)/antibodies against CD146 (FITC/green) (for EC) and CD133 (for PC/rhodamine/red) (EPC, CD146+/CD133+/DAPI+). Cells indicated with arrows are EPCs (A–D) from mice treated with PBS (A: Control), NT (B), METH (C), and gp120 (D). (TIF) [file pone.0062164.s001.tif]
